# Participation of UV-regulated Genes in the Response to Helix-distorting DNA Damage in the Thermoacidophilic Crenarchaeon *Sulfolobus acidocaldarius*

**DOI:** 10.1264/jsme2.ME19055

**Published:** 2019-12-27

**Authors:** Shoji Suzuki, Norio Kurosawa

**Affiliations:** 1 Department of Science and Engineering for Sustainable Development, Faculty of Science and Engineering, Soka University Tokyo Japan

**Keywords:** *Sulfolobus*, gene knockout, response to UV irradiation, helix-distorting DNA lesion, DNA repair

## Abstract

Several species of *Sulfolobales* have been used as model organisms in the study of response mechanisms to ultraviolet (UV) irradiation in hyperthermophilic crenarchaea. To date, the transcriptional responses of genes involved in the initiation of DNA replication, transcriptional regulation, protein phosphorylation, and hypothetical function have been observed in *Sulfolobales* species after UV irradiation. However, due to the absence of knockout experiments, the functions of these genes under *in situ* UV irradiation have not yet been demonstrated. In the present study, we constructed five gene knockout strains (*cdc6-2*, *tfb3*, *rio1*, and two genes encoding the hypothetical proteins, Saci_0951 and Saci_1302) of *Sulfolobus acidocaldarius* and examined their sensitivities to UV irradiation. The knockout strains exhibited significant sensitivities to UV-B irradiation, indicating that the five UV-regulated genes play an important role in responses to UV irradiation *in vivo*. Furthermore, Δ*cdc6-2*, Δ*rio1*, ΔSaci_0951, and Δ*tfb3* were sensitive to a wide variety of helix-distorting DNA lesions, including UV-induced DNA damage, an intra-strand crosslink, and bulky adducts. These results reveal that *cdc6-2*, *tfb3*, *rio1*, and Saci_0951 are play more important roles in broad responses to helix-distorting DNA damage than in specific responses to UV irradiation.

Members of the order *Sulfolobales* belonging to the class *Crenarchaeota* are distributed in hot acidic terrestrial environments (39 and references therein). Seven genera belonging to the order *Sulfolobales* have been described with validly published names: *Sulfolobus* (6), *Acidianus* (44), *Metallosphaera* (23), *Stygiolobus* (45), *Sulfurisphaera* (26, 50), *Sulfodiicoccus* (38), and *Saccharolobus* (39). *Sulfolobus solfataricus*, which has been intensively investigated in biochemical and genetic studies, has been reclassified as *Saccharolobus solfataricus* (39). Most members of the order *Sulfolobales* are aerobic and acidophilic (hyper-)thermophiles (46). They have the ability to adapt to various conditions and damage, such as hydrolytic reactions, aerobic conditions, and acidic conditions (14, 29, 57). Unlike hyperthermophilic archaea living in deep-sea hydrothermal vents (47), members of the order *Sulfolobales* are exposed to UV irradiation (14). Despite these extreme conditions, members of the order *Sulfolobales* are often dominant in acidic geothermal areas worldwide (31, 55), suggesting the presence of efficient mechanisms of adaptation to harsh conditions. Therefore, studies that provide insights into the molecular mechanisms underlying this adaptation are crucial for understanding the survival of *Sulfolobales* in these extreme habitats.

The denaturation and degradation of nucleic acids are accelerated under high temperatures (16, 29). Thus, (hyper-) thermophilic archaea harbor multiple strategies to maintain their nucleic acids, such as heat-protective DNA chaperone activity by reverse gyrase and efficient DNA repair systems (4, 15–19, 24). Furthermore, acidophilic (hyper-)thermophiles adapt to accelerated oxidative stress under acidic conditions. They also control cytoplasmic pH to protect nucleic acids from oxidative stress (1, 32). However, the DNA repair mechanisms of oxidative bases remain unclear.

In contrast to thermal and oxidative stresses, UV irradiation generally produces photoproducts (*i.e.*, cyclobutane pyrimidine dimers [CPDs] and [6–4] photoproducts) as helix-distorting DNA lesions, and unrepaired photoproducts become the source of mutations (36, 57). Moreover, a double-stranded DNA break (DSB) is formed as a result of the collision between the replication fork and unrepaired CPD (11), which causes mortal damage. An integrated model of UV-specific reactions in *Sulfolobus* and *Saccharolobus* was reported by Fröls *et al.* (12). In brief, this model involves the following: i) CPDs are removed from genomic DNA by DNA photolyase and an unidentified repair pathway (12, 18); ii) as a possible secondary effect, DNA lesions (*i.e.*, DSBs) cause cell cycle arrest and repress the initiation of replication until repair may be completed; and iii) Ups pili are induced and mediate the aggregation of cells, enhancing the chance of DNA repair through homologous recombination by the crenarchaeal system for the exchange of DNA (Ced system) (58, 59). Thus far, a more detailed process has been described (12), the effects of DNA damage on cell cycle progression (DNA content distribution) in *Sulfolobales* species has been reported (11, 14, 20), and genetic analyses of DNA repair involved in UV damage in hyperthermophilic archaea have been presented and reviewed (13, 18, 57, 62). However, numerous new questions have been raised.

Two previous DNA microarray analyses revealed a transcriptional response to UV irradiation in two species of the order *Sulfolobales*, namely, *S. solfataricus* and *S. acidocaldarius* (11, 14), simultaneously raising further questions. These studies reported that DNA repair proteins were not induced. However, clear transcriptional responses were observed with the down-regulation of the DNA replication machinery, changes in transcriptional regulatory proteins, and the up-regulation of protein kinase and conserved hypothetical proteins in the *Sulfolobales* species. Among these genes, we herein focused on *cdc6-2*, *tfb3*, *rio1*, Saci_0951, and Saci_1302. These genes, except for *rio1*, are regulated under UV irradiation with a conserved UV-regulated promoter hexanucleotide motif (5′-ANTTTC-3′) (28). The *cdc6-2* gene encodes a protein that may act as a repressor of replication initiation, whereas Cdc6-1 and Cdc6-3 act in a positive manner (37). UV irradiation affects the balance between the repressor and enhancers, and the dominance of the repressor Cdc6-2 under UV irradiation may be required to repress the initiation of a new round of DNA replication, thereby allowing sufficient time for DNA repair (14). *S. acidocaldarius* encodes three homologues of the *tfb* (transcription factor 2B) gene (7, 14). The *tfb3* gene is one of most highly up-regulated transcripts in response to UV irradiation in *S. acidocaldarius* (14) and is considered to be required to modulate transcription from general promoters (14, 33). The *rio1* gene encodes Ser-/Thr-RIO protein kinase-1 and is induced in *S. solfataricus* and *S. acidocaldarius* after UV irradiation (14), whereas the biological targets of RIO kinase remain unknown. Two genes encoding the hypothetical proteins Saci_0951 and Saci_1302 (found only in *Sulfolobales*) are strongly induced in *S. acidocaldarius* following UV irradiation (14). These UV-regulated genes participate in responses to UV irradiation. However, there is currently no evidence to show the participation of these regulated genes in the responses of the hyperthermophilic archaea *Sulfolobales* species to UV irradiation *in vivo*.

The use of gene knockout in genetic studies is a powerful analytical method for answering questions arising from previous studies (11, 14). To date, the gene knockout approach has been employed in four species of *Sulfolobales*: *S. acidocaldarius* (40, 49, 50, 53, 54), *S. solfataricus* (2), *S. islandicus* (8, 34, 61–64), and *Metallosphaera sedula* (30). We recently constructed the DNA photolyase and restriction endonuclease *Sua*I-deficient *S. acidocaldarius* strain DP-1 (Δ*pyrE* Δ *suaI* Δ*phr*) as a host strain for use in a genetic study (50). To understand the *in situ* functions of proteins encoded in *cdc6-2*, *tfb3*, *rio1*, Saci_0951, and Saci_1302, we constructed respective gene knockout strains of *S. acidocaldarius* using the PCR tailing method previously reported (40). The PCR tailing method is a knockout technique in which the target gene is replaced with a selectable marker gene through double-crossover events. The characteristics of the knockout strains after UV-B irradiation (*i.e.*, growth property, survival, and repair of CPDs) were examined. UV-regulated genes were found to be required for responses not only to UV-induced DNA lesions, but also to other types of DNA damage. We then discussed the sensitivities of the knockout strains to other helix-distorting DNA lesions (*i.e.*, an intra-strand crosslink and bulky adducts) and characterized the mutant phenotypes.

## Materials and Methods

### Strains and growth conditions

The strains used in the present study are listed in Table S1. The *S. acidocaldarius* pyrimidine auxotrophic, restriction endonuclease *Sua*I and DNA photolyase (Phr)-deficient strain DP-1 (Δ*pyrE* Δ*suaI* Δ*phr*) were constructed from the strain SK-1 (Δ*pyrE* Δ*suaI*) using pop-out recombination (50). DP-1 was then used as the host and parent strain in the present study. DP-1 and its derivatives were cultivated in xylose and tryptone (XT) medium (pH 3) at 75°C with or without shaking at 160 rpm, as previously described (35). To grow the pyrimidine auxotrophic strain, 0.02 g L^−1^ of uracil was added to XT medium (XTU). Solid plate medium was prepared as previously described (50).

### General DNA manipulation

General DNA manipulation was conducted as previously described (50).

### Construction of gene knockout strains

Gene knockout strains of *S. acidocaldarius* were constructed through the electrotransformation of knockout cassettes, which were prepared via one-step construction (PCR tailing) followed by the isolation of transformants.

The plasmid and linear DNA used in the present study are shown in Table S1, and the PCR primers (*i.e.*, KO primers and outer primers) used are listed in Table S2. The PCR tailing method was utilized for the knockout of five genes, namely, *cdc6-2* (Saci_0903), *tfb3* (Saci_0665), *rio1* (Saci_0965), and two hypothetical genes (Saci_0951 and Saci_1302). In brief, the *cdc6-2* knockout PCR product (Cdc6-2-knock) was amplified from placSpyrE (Table S1) as a template using the primers Cdc6-2-KO-F/R (containing 48 bp of the 5′ and 3′ flanking regions of *cdc6-2*) and Premix Taq Ex Taq (version 2.0) (Takara Bio, Kusatsu, Japan) as the DNA polymerase. Similarly, the other knockout PCR products, *i.e.*, TFB3-knock, RIO-knock, Saci_0966-knock, Saci_0951-knock, Saci_0949-knock, Saci_0949–0950-knock, and Saci_1302-knock, were amplified using the primers TFB3-KO-F/R, RIO-KO-F/R, Saci_0966-KO-F/R, Saci_0951-KO-F/R, Saci_0949-KO-F/Saci_0949–0950-KO-R, Saci_0949–0950-KO-F/R, and Saci_1302-KO-F/R, respectively. The purified PCR products were used for transformation based on electroporation, which facilitates DNA entry into cells via electrically induced membrane pores (43, 60).

The preparation of a competent cell and the transformation procedure for gene knockout have already been described (50). Gene knockout was conducted using an optimized transformation protocol (50).

X-gal staining was performed by spotting 1 μL of X-gal solution (10 mg mL^−1^ of 0.85% NaCl_2_) onto colonies and incubating at 75°C for 1 d. In contrast to the non-transformants (wild-type *S. acidocaldarius*), the transformants (*lacS*^+^) converted the chemical into a strong blue substance (5).

### Growth curve after UV irradiation

One milliliter of each overnight culture of the parent strain DP-1, Δ*cdc6-2*, Δ*tfb3*, Δ*rio1*, ΔSaci_0951, and ΔSaci_1302 (late-log to stationary phase) was poured into 90×15 mm plastic Petri dishes (AGC TECHNO GLASS, Yoshida-cho, Shizuoka, Japan) and then exposed to UV-B irradiation using a UV lamp (UVM-57, 302 nm, 6 W; Analytik Jena AG, Jena, Germany) positioned 6.5 cm above the top of the dish at room temperature for 0, 20, 40, and 60 s (yield: 0, 400, 800, and 1,200 J m^−2^, respectively). An irradiated sample was collected after every 20 s of irradiation and inoculated into three test tubes containing 6 mL of XT or XTU liquid medium to yield an initial OD_600_=0.005 (triplicates). Cells were subsequently cultivated at 75°C with shaking at 160 rpm. Thereafter, cell growth was monitored by measuring OD_600_.

### Growth curve in the presence of DNA-damaging agents and antibiotics

The chemicals used in the present study are listed in Table 1. Each overnight culture of the knockout strains (late-log to stationary phase) was inoculated into 6 mL of XT or XTU liquid medium containing DNA-damaging agents (cisplatin [Wako, Osaka, Japan] [0, 20, 30, and 40 μg mL^−1^], 4-nitroquinoline N-oxide (4-NQO) [Wako] [0, 0.3, 0.4, and 0.5 μg mL^−1^], and metronidazole [TCI, Tokyo, Japan] [0, 0.4, 0.8, and 1.2 mg mL^−1^]) and antibiotics (actinomycin D [Nacalai Tesque, Kyoto, Japan] [0, 2, 4, and 5 μg mL^−1^] and novobiocin [Nacalai Tesque] [0, 1, 2, and 3 μg mL^−1^]) to yield an initial OD_600_=0.005. Cells were subsequently cultivated at 75°C with or without shaking for the growth curve in the presence of DNA-damaging agents and antibiotics. Thereafter, cell growth was monitored by measuring the OD_600_.

### UV survival test using the spotting assay

One milliliter of each overnight culture of the parent strain (OD_600_=0.800), Δ*cdc6-2* (OD_600_=0.845), Δ*tfb3* (OD_600_=0.806), Δ*rio1* (OD_600_=0796), ΔSaci_0951 (OD_600_=0.628), and ΔSaci_1302 (OD_600_=0.825) (late-log to stationary phase) was poured into 90×15 mm plastic petri dishes (AGC TECHNO GLASS) and irradiated with UV light at room temperature for 0, 20, 40, and 60 s (yield: 0, 400, 800, and 1,200 J m^−2^, respectively). Irradiated samples (approximately 100 μL each) were collected with every 20 s of irradiation. Subsequently, 20 mM of sucrose was used for the preparation of proper diluted samples (10^0^–10^−6^), and 5 μL of the diluted samples was spotted on the XTU plates. Samples were vortexed for 5 s prior to the dilution and spotting of samples. Plates were incubated at 75°C for 6 d.

### Analysis of DNA repair properties of CPDs

A method for the analysis of the repair properties of CPDs was previously reported by Salerno *et al.* (43). Each *S. acidocaldarius* strain of the culture of the parent strain, Δ*cdc6-2*, Δ*tfb3*, Δ*rio1*, ΔSaci_0951, and ΔSaci_1302, was irradiated with UV light (1,200 J m^−2^) or with a mock treatment (*i.e.*, without UV irradiation). After UV irradiation, these strains were cultivated at 75°C. Genomic DNA was extracted after 0–25 h using a Magtration System 12GC (Precision System Science, Matsudo, Japan). Extracted genomic DNA was digested using T4 Endonuclease V (T4 EndoV) (New England Biolabs, Ipswich, MA, USA) at 37°C for 30 min, according to the instructions provided by the manufacturer. Ten units of enzyme were utilized per 100 ng of genomic DNA. Samples were then denatured at 85°C for 3 min and incubated at 4°C for 3 min. Genomic bands were monitored using 1% agarose gel staining with ethidium bromide.

## Results

### Construction of gene knockout strains

**Fig. 1:**
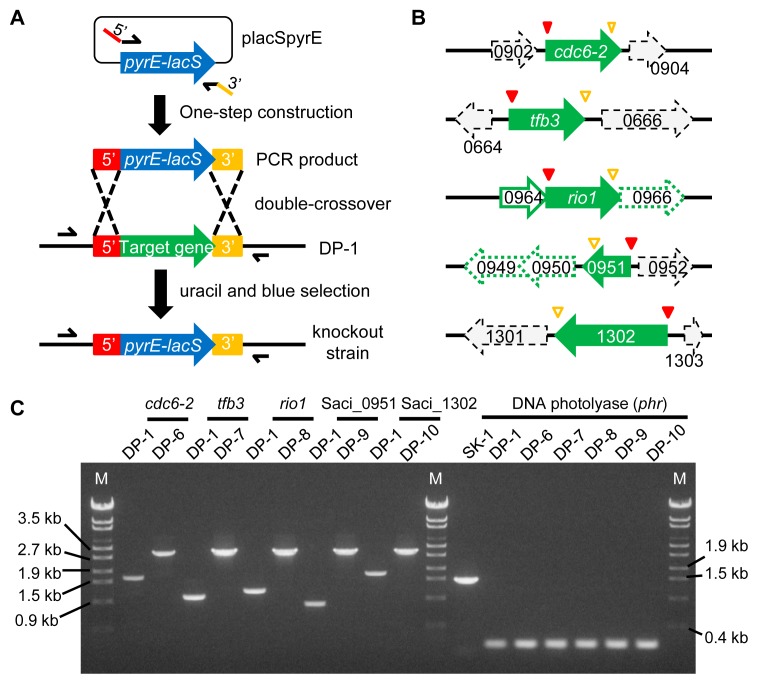
Schematic of gene knockout. (**A**) Construction of gene knockout mutants based on the PCR tailing method. Knockout PCR products were generated by one-step construction using primers attached at the 5′ and 3′ homologous regions of the target gene to the 5′ ends. PCR products were electropolated into the strain DP-1 (Δ*pyrE* Δ*suaI* Δ*phr*). A double-crossover between the PCR product and chromosome at the 5′ and 3′ regions resulted in the replacement of the target gene with the *pyrE-lacS* marker. The resulting uracil prototroph transformants exhibited blue colonies and were selected on uracil-free plates. Arrows show the positions of outer PCR primers. (**B**) Setting 5′ and 3′ regions of target genes. Green arrows indicate target genes, arrows with a green frame (thick solid) are genes in the same operon with the target gene, while thin arrows with a black-dotted frame are not. Arrows with a green-dotted thick frame may be genes forming an operon with the target gene. Red and yellow-framed triangles represent the 5′ and 3′ homologous regions, respectively. The number indicates the gene number of Saci_. (**C**) PCR analysis of target gene loci (*cdc6-2*, *tfb3*, *rio1*, Saci_0951, and Saci_1302) and the DNA photolyase (*phr*) locus of the *S. acidocaldarius* strains DP-1, DP-6, DP-7, DP-8, DP-9, DP-10, and SK-1 using outer primers. The expected sizes of PCR bands were as follows: 1.6 kb (wt) and 2.9 kb (mutant) in the *cdc6-2* locus; 1 kb (wt) and 2.9 kb (mutant) in the *tfb3* locus; 1.2 kb (wt) and 2.9 kb (mutant) in the *rio1* locus; 0.8 kb (wt) and 2.9 kb (mutant) in the Saci_0951 locus; 1.7 kb (wt) and 2.9 kb (mutant) in the Saci_1302 locus; 1.5 kb (wt) and 0.2 kb (mutant) in the *phr* locus. A λ-EcoT14 was loaded in lane M.

The PCR tailing method (40) was performed to construct the *cdc6-2*-, *tfb3*-, *rio1*-, Saci_0951-, and Saci_1302-deficient strains. To disrupt the target genes, knockout cassettes composed of a *pyrE-lacS* dual marker that attaches two 48-bp 5′ and 3′ homologous arms to the ends of the marker were constructed using one-step PCR (*i.e.*, Cdc6-2-knock, TFB3-knock, RIO kinase-knock, Saci_0951-knock, and Saci_1302-knock) (Fig. 1A and B). After 2 μg of knockout cassettes were electroporated (15 kV cm^−1^, 9 ms) into the DNA photolyase *phr*deficient strain DP-1 (Δ*pyrE* Δ *suaI* Δ *phr*) harvested at the late-log phase (OD_600_=0.532), colonies grew on XT plates for 5 d of cultivation (*i.e.*, 1 colony μg^−1^ Cdc6-2-knock, 8 colonies μg^−1^ TFB3-knock, 2 colonies μg^−1^ RIO kinase-knock, 9 colonies μg^−1^ Saci_0951-knock, and 2 colonies μg^−1^ Saci_1302). Two to three colonies were then selected, and blue selection was performed by spotting 1 μL of the X-gal solution. After blue selection at 75°C for 1 d, the selected colonies were stained blue (data not shown). Two blue colonies of each knockout strain were purified via single colony isolation and analyzed with PCR screening using outer primers (Fig. 1A). The band sizes of these colonies were identical to those obtained for the expected transformants carrying the insertion of a 2.5-kb *pyrE-lacS* marker into the target gene locus (Fig. 1C). Each correct strain of Δ*cdc6-2*, Δ*tfb3*, Δ*rio1*, ΔSaci_0951, and ΔSaci_1302 was designated as the *S. acidocaldarius* strains DP-6 (Δ*suaI* Δ *phr* Δ *cdc6-2*::*pyrE*-*lacS*), DP-7 (Δ*suaI* Δ *phr* Δ*tfb3*::*pyrE*-*lacS*), DP-8 (Δ*suaI* Δ *phr* Δ *rio1*::*pyrE-lacS*), DP-9 (Δ*suaI* Δ *phr* ΔSaci_0951::*pyrE-lacS*), and DP-10 (Δ*suaI* Δ*phr* ΔSaci_1302::*pyrE-lacS*), respectively, and used in the phenotypic analysis. The deletion of the *phr* (encoding DNA photolyase) locus was confirmed through a PCR analysis using the outer primers (Fig. 1C), indicating that these knockout strains had a 1.3-kb deletion in the *phr* locus.

### Growth curves of knockout strains after UV-B irradiation

**Fig. 2:**
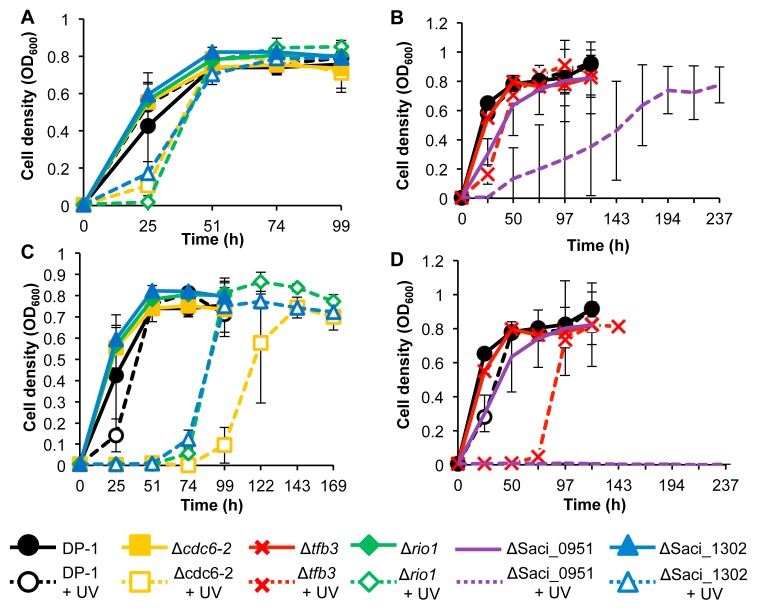
Growth properties of knockout strains after UV irradiation. Each overnight culture of DP-1 (parent strain), Δ*cdc6-2*, Δ*tfb3*, Δ*rio1*, ΔSaci_0951, and ΔSaci_1302 was exposed to UV light (0 [**A–D**], 400 [**A**, **B**], and 800 J m^−2^ [**C**, **D**], respectively, +UV represents a UV-treated sample) followed by an inoculation in liquid medium and cultivation at 75°C with shaking. Growth curves of Δ*cdc6-2*, Δ*rio1*, and ΔSaci_1302 were indicated in (**A** and **C**), those of Δ*tfb3* and ΔSaci_0951 were shown in (**B** and **D**). Data are means±SD calculated using three biological replicates.

The DNA photolyase-deficient strain DP-1 and its derivatives did not exhibit photoreactivation activity under light conditions (50). To characterize the UV sensitivity of the knockout strains Δ*cdc6-2*, Δ*tfb3*, Δ*rio1*, ΔSaci_0951, and ΔSaci_1302, we examined their growth properties in parallel with the parent strain DP-1 after UV-B irradiation (0, 400, and 800 J m^−2^) (Fig. 2). The generation times and final cell concentrations of Δ*cdc6-2*, Δ*tfb3*, Δ*rio1*, and ΔSaci_1302 were similar to those of DP-1 without UV irradiation (Fig. 2A and B). In contrast, the growth rate of ΔSaci_0951 was slightly slower than that of DP-1 (Fig. 2B). When DP-1 was exposed to UV irradiation at 400 J m^−2^ (Fig. 2A and B), growth was identical to that of mock-treated DP-1 (without UV irradiation). The growth of Δ*cdc6-2*, Δ*tfb3*, Δ*rio1*, and ΔSaci_1302 was slightly retarded after UV irradiation at 400 J m^−2^ (Fig. 2A and B), whereas that of ΔSaci_0951 was markedly delayed (Fig. 2B). After UV irradiation at 800 J m^−2^, the growth of Δ*cdc6-2*, Δ*tfb3*, Δ*rio1*, and ΔSaci_1302 was delayed significantly more than that of DP-1 (Fig. 2C and D). We did not observe the growth of ΔSaci_0951 after exposure to UV light at 800 J m^−2^ (Fig. 2D). Thus, our results clearly showed that the knockout strains Δ*cdc6-2*, Δ*tfb3*, Δ*rio1*, ΔSaci_0951, and ΔSaci_1302 were more sensitive to UV-B irradiation than its parent strain.

### Characterization of UV sensitivities of knockout strains using the spotting assay

**Fig. 3:**
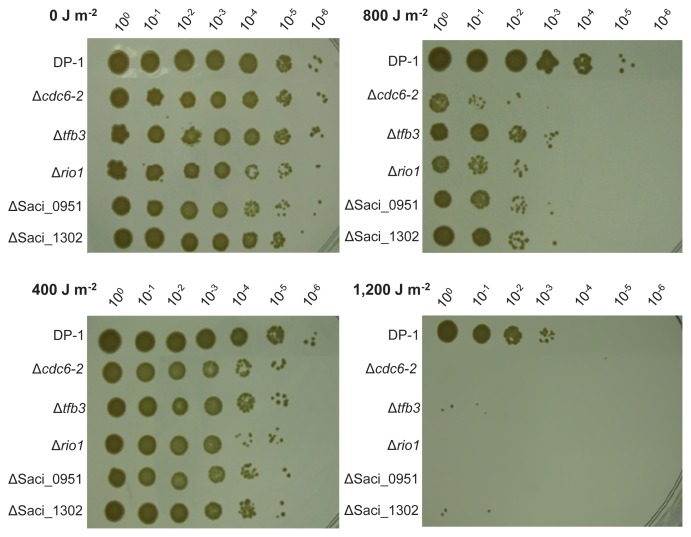
Analysis of UV survival of knockout strains by the spotting test. Each overnight culture of DP-1 (parent strain), Δ*cdc6-2*, Δ*tfb3*, Δ*rio1*, ΔSaci_0951, and ΔSaci_1302 was irradiated with UV light (0, 400, 800, and 1,200 J m^−2^, respectively), and aliquots were serially diluted (10^0^–10^−6^) and spotted on XTU plates. Plates were cultivated at 75°C. Experiments were repeated three times with similar results.

The UV-B survival of the knockout strains Δ*cdc6-2*, Δ*tfb3*, Δ*rio1*, ΔSaci_0951, and ΔSaci_1302 was examined and compared with that of the parent strain DP-1 (Fig. 3). Colony growth observed in mock-treated knockout strains was similar to that of DP-1 (0 J m^−2^). After UV irradiation at 400 J m^−2^, Δ*cdc6-2*, Δ*tfb3*, Δ*rio1*, ΔSaci_0951, and ΔSaci_1302 exhibited slightly stronger sensitivity to UV irradiation than DP-1. These sensitivities toward UV irradiation were accelerated at higher UV dosages (800 and 1,200 J m^−2^). Notably, following the exposure of DP-1 to UV light at 1,200 J m^−2^, most of the cells survived. In contrast, colonies of the knockout strains hardly grew after UV irradiation at 1,200 J m^−2^. These results demonstrated that Δ*cdc6-2*, Δ*tfb3*, Δ*rio1*, ΔSaci_0951, and ΔSaci_1302 were significantly sensitive to UV-B irradiation.

### UV-regulated genes are not essential for the DNA repair of UV-induced DNA photoproducts

**Fig. 4:**
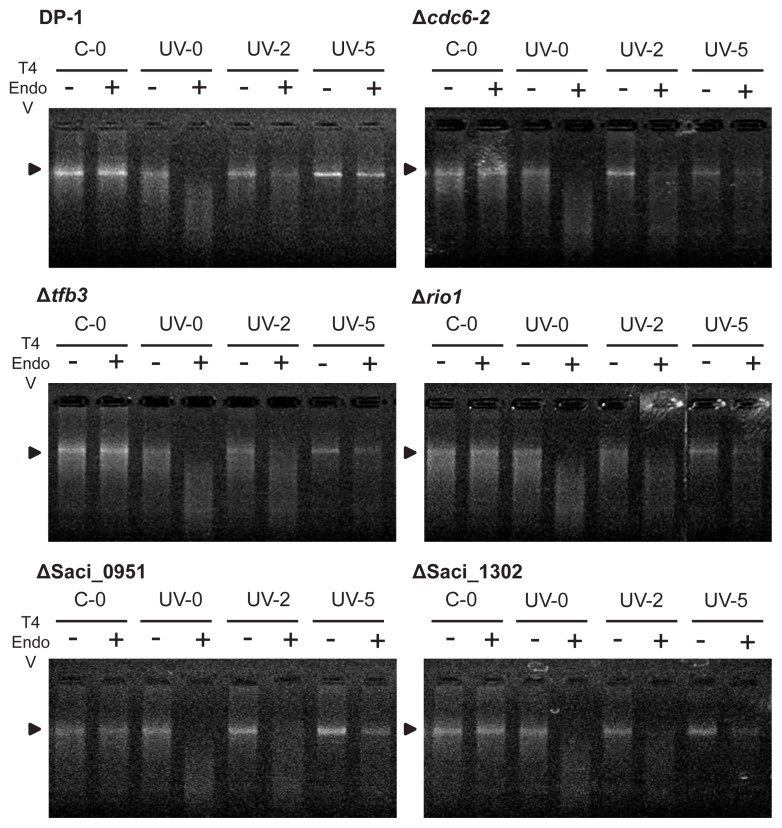
Analysis of DNA repair properties of cyclobutane pyrimidine dimers (CPDs) in knockout strains shown by gel electrophoresis. Genomic DNA extracted from cells grown in liquid medium after UV irradiation. The mock-treated sample (without UV irradiation) was shown by C-0. Numbers (0, 2, and 5) indicate h after UV irradiation (UV). Lanes “+” and “−” indicate the presence or absence of T4 EndoV digestion, respectively. Genomic DNA was denatured and loaded on a 1% agarose gel stained with ethidium bromide. Arrows indicate the position of uncut genome bands.

The repair properties of CPDs were characterized according to the experiment described by Salerno *et al.* (42). This approach involved a CPD-specific digestion assay; however, the denaturation of genomic DNA was conducted at a high temperature. To clarify the capacity for the dark repair of CPDs as UV-induced DNA lesions, the parent strain (DP-1) and knockout strains Δ*cdc6-2*, Δ*tfb3*, Δ*rio1*, ΔSaci_0951, and ΔSaci_1302 were exposed to UV light (1,200 J m^−2^) and immediately cultivated at 75°C. In this experiment using *phr*-deficient strains, CPDs were repaired through unknown DNA repair mechanisms (42) rather than through photoreactivation, even under light conditions. Genomic DNA was isolated from the cells at various time points after UV irradiation and treated with T4 EndoV, an enzyme that specifically introduces a nick at the CPD site. Denatured genomic DNA was subsequently monitored using agarose gel electrophoresis (Fig. 4). Genomic DNA extracted from the mock-treated cultures of DP-1, Δ*cdc6-2*, Δ*tfb3*, Δ*rio1*, ΔSaci_0951, and ΔSaci_1302 was not digested (lane C-0+), whereas that of the exposed samples was cleaved (lane UV-0+). When cells were cultivated at 75°C for 2 h after UV irradiation, some of the genomic DNA of DP-1 and Δ*cdc6-2* was not digested (lane UV-2+), indicating that DNA repair had already been initiated. Similarly, DNA repair was initiated in Δ*tfb3*, Δ*rio1*, ΔSaci_0951, and ΔSaci_1302, as indicated by the presence of longer DNA observed after 2 h of UV irradiation (lane UV-2+). The majority of DNA lesions were removed in DP-1 within 5 h (lane UV-5+). Uncut genomic DNA bands were observed in all knockout strains within 5 h (lane UV-5+). These results revealed that Cdc6-2, TFB3, RIO1, Saci_0951, and Saci_1302 were not essential in the DNA repair of CPDs in the thermophilic crenarchaeon *S. acidocaldarius*.

### Sensitivity of knockout strains to helix-distorting DNA lesions

**Fig. 5:**
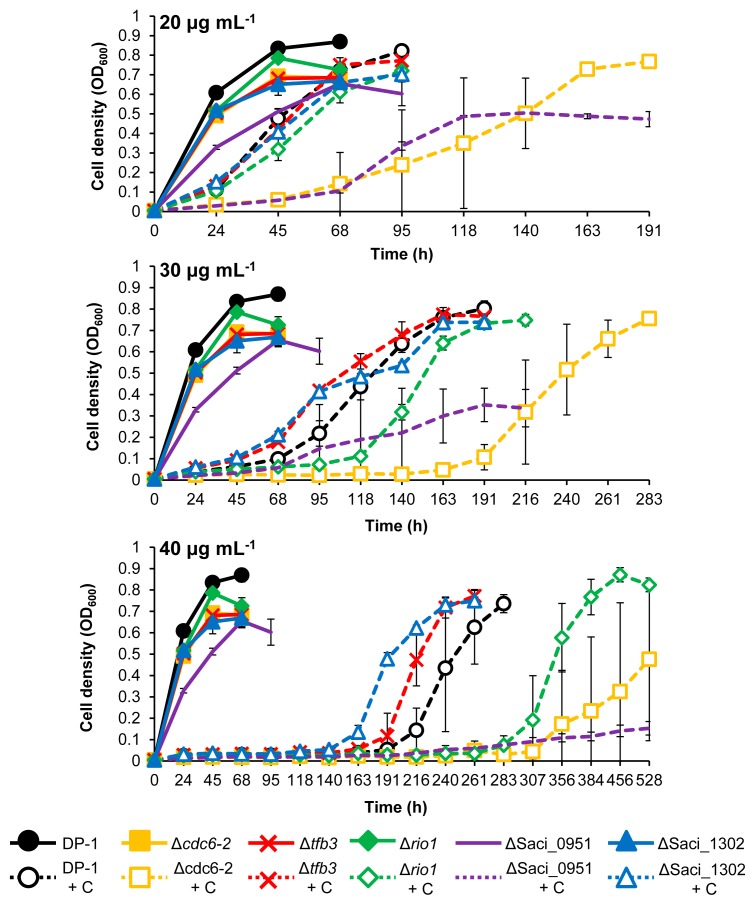
Growth properties of knockout strains in the presence of cisplatin. Each overnight culture of DP-1, Δ*cdc6-2*, Δ*tfb3*, Δ*rio1*, ΔSaci_0951, and ΔSaci_1302 was inoculated in liquid medium and cultivated at 75°C with shaking (+C represents growth in the presence of 20, 30, and 40 μg mL^−1^ cisplatin, respectively). Data are means±SD calculated using three biological replicates.

**Fig. 6:**
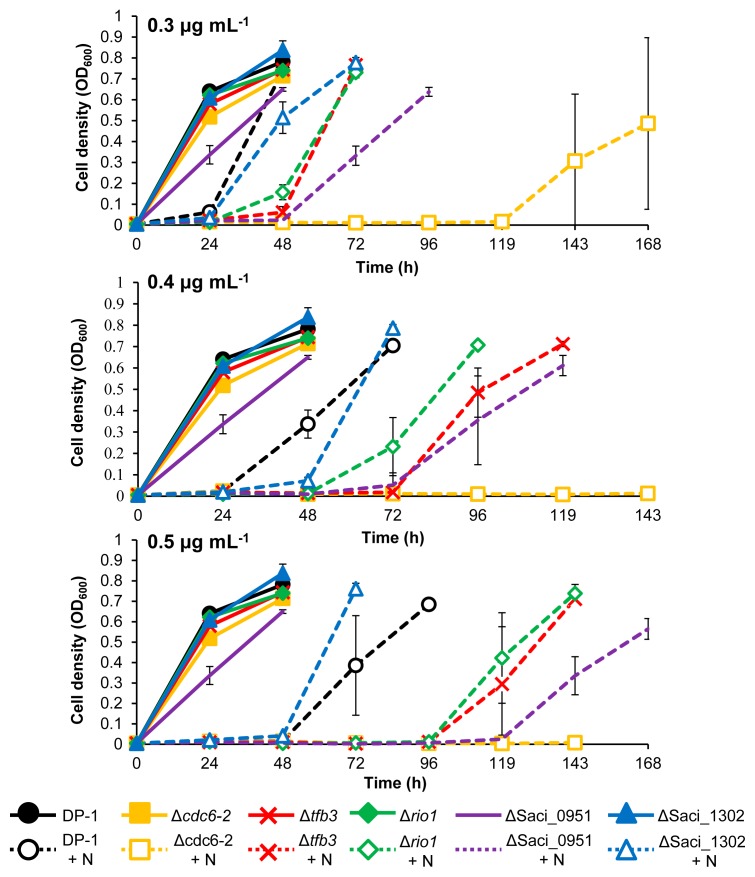
Growth properties of knockout strains in the presence of 4-nitroquinoline N-oxide (4-NQO). Each overnight culture of DP-1, Δ*cdc6-2*, Δ*tfb3*, Δ*rio1*, ΔSaci_0951, and ΔSaci_1302 was inoculated in liquid medium and cultivated at 75°C with shaking (+N represents growth in the presence of 0.3, 0.4, and 0.5 μg mL^−1^ 4-NQO, respectively). Data are means±SD calculated using three biological replicates.

**Fig. 7:**
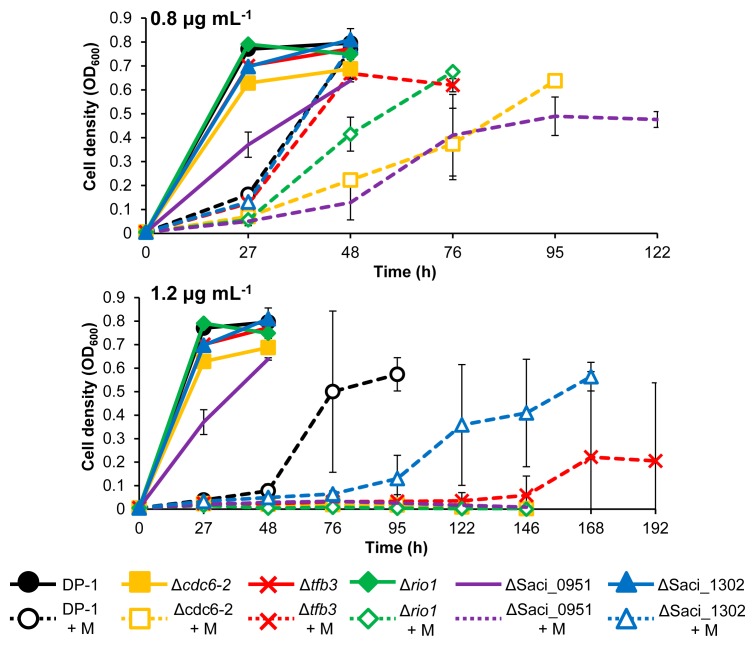
Growth properties of knockout strains in the presence of metronidazole. Each overnight culture of DP-1, Δ*cdc6-2*, Δ*tfb3*, Δ*rio1*, ΔSaci_0951, and ΔSaci_1302 was inoculated in liquid medium and cultivated at 75°C with shaking (+M represents growth in the presence of 0.8 and 1.2 mg mL^−1^ metronidazole, respectively). Data are means±SD calculated using three biological replicates.

CPDs are helix-distorting DNA lesions (56). We investigated whether UV-regulated genes are involved in the response or DNA repair of UV-induced DNA damage as well as other types of helix-distorting DNA lesions. To examine the sensitivities of knockout strains to the intra-strand crosslink (cisplatin) and bulky adducts (4-NQO and metronidazole) (41), the growth properties of the knockout strains were characterized in the presence or absence of helix-distorting DNA-damaging agents and compared with the parent strain DP-1 (Fig. 5, 6, and 7).

The cisplatin sensitivity test showed that the growth of Δ*tfb3* and ΔSaci_1302 was similar to that of DP-1 in the presence of 20 and 30 μg mL^−1^ cisplatin. However, their growth was slightly faster than that of DP-1 in the presence of 40 μg mL^−1^ cisplatin (Fig. 5). Similarly, no marked difference was observed between the growth of Δ*rio1* and that of DP-1 in the presence of 20 μg mL^−1^ cisplatin. Conversely, the growth of Δ*rio1* was delayed more than that of DP-1 in the presence of higher concentrations of cisplatin (30 and 40 μg mL^−1^). The significant growth retardation of Δ*cdc6-2* and ΔSaci_0951 was observed in the presence of 20–40 μg mL^−1^ cisplatin versus the growth of DP-1. Furthermore, the maximum cell density and rate of ΔSaci_0951 gradually decreased with increasing concentrations of cisplatin. These results clearly indicated that Δ*cdc6-2*, Δ*rio1*, and ΔSaci_0951 exhibited strong sensitivities to DNA damage caused by cisplatin as an intra-strand crosslink DNA agent.

The 4-NQO sensitivity test indicated that the growth property of ΔSaci_1302 was similar to that of DP-1 in the presence of 0.3–0.5 μg mL^−1^ 4-NQO (Fig. 6). In the presence of 0.3–0.5 μg mL^−1^ 4-NQO, we observed the growth retardation of Δ*tfb3*, Δ*rio1*, and ΔSaci_0951 versus DP-1. The growth of Δ*cdc6-2* was delayed significantly more in the presence of 0.3 μg mL^−1^ 4-NQO than that of DP-1. In addition, Δ*cdc6-2* did not grow in the presence of higher concentrations of 4-NQO (*i.e.*, 0.4 and 0.5 μg mL^−1^). These results revealed that Δ*tfb3*, Δ*rio1*, and ΔSaci_0951 were sensitive to DNA adducts caused by 4-NQO and that Δ*cdc6-2* exhibited strong sensitivity.

The metronidazole sensitivity test did not demonstrate the significant growth retardation of the five knockout strains in the presence of 0.4 mg mL^−1^ metronidazole from that of DP-1 (data not shown). The growth of Δ*tfb3* and ΔSaci_1302 was identical to that of DP-1 in the presence of 0.8 mg mL^−1^ metronidazole. Moreover, the growth of Δ*tfb3* and ΔSaci_1302 was delayed more than that of DP-1 in the presence of 1.2 mg mL^−1^ metronidazole (Fig. 7). The growth retardation of Δ*cdc6-2*, Δ*rio1*, and ΔSaci_0951 was observed in the presence of 0.8 mg mL^−1^ metronidazole. Furthermore, Δ*cdc6-2*, Δ*rio1*, and ΔSaci_0951 did not grow in the presence of 1.2 mg mL^−1^ metronidazole. These results demonstrated that Δ*tfb3* and ΔSaci_1302 were sensitive to the DNA adducts caused by metronidazole and that Δ*cdc6-2*, Δ*rio1*, and ΔSaci_0951 were significantly sensitive.

After UV irradiation, DNA replication in *S. acidocaldarius* and *S. solfataricus* is repressed (14). To investigate the participation of UV-regulated genes in cell cycle regulation (DNA replication process), we examined the sensitivities of the knockout strains to DNA replication inhibitors (*i.e.*, actinomycin D and novobiocin) (21). However, this experiment did not reveal significant growth retardation in the knockout strains versus DP-1 (data not shown).

## Discussion

Two independent studies on transcriptional responses to UV irradiation in *S. acidocaldarius* and *S. solfataricus* and a subsequent analysis revealed that the Ups pilus is a DNA transfer system for the exchange of DNA following DNA damage (11, 14, 58). However, several important aspects of UV-regulated genes in the *Sulfolobales* species have not yet been investigated (14). The present study aims to provide critical experimental evidence of the participation of UV-regulated genes (*cdc6-2*, *tfb3*, *rio1*, Saci_0951, and Saci_1302) in the *in vivo* responses of the thermophilic crenarchaeon *S. acidocaldarius* to UV irradiation. In addition to the Ups pili as a UV-responsive system (58), our genetic study revealed that five UV-regulated genes are involved in the *in vivo* response to UV irradiation. *S. acidocaldarius* clearly responds to UV-induced DNA damage at the transcriptional level. In addition, we demonstrated the involvement of *cdc6-2*, *tfb3*, *rio1*, and Saci_0951 in the responses to a variety of helix-distorting DNA lesions. This evidence expands our knowledge on the responses of the model crenarchaea *Sulfolobus* to DNA damage.

Based on the abundance of Cdc6 proteins in the cell cycle and binding patterns, Cdc6-2 may act as an inhibitor of the initiation of DNA replication (37). The initiation of DNA replication is inhibited in *S. acidocaldarius* after UV irradiation (14). Based on the present results (Fig. 2 and 3) and previous findings (14, 37), the *cdc6-2*-deficient strain may exhibit sensitivity to UV irradiation because Δ*cdc6-2* is unable to inhibit the initiation of DNA replication after UV irradiation.

Our results (Fig. 2 and 3) revealed the participation of TFB3 in the *in vivo* responses to UV irradiation. These results suggest that the modulation of the transcriptional response by TFB3 is essential for survival after UV irradiation, particularly in cellular processes, rather than the DNA repair of UV-induced lesions. This may be attributed to the capability of Δ*tfb3* for the DNA repair of CPDs. A recent study revealed that TFB3 plays an essential role in regulating the number of genes involved in cell aggregation (*ups* operon) and/or intercellular DNA exchange (*ced* system) in *S. islandicus* (10).

The *in vivo* functions of eukaryotic RIO kinases have been characterized in detail, whereas its prokaryotic RIO kinases have not (9, 27). For example, eukaryotic RIO kinase is essential in cell cycle progression and chromosome maintenance, and is also required for the synthesis of new ribosomes (3, 52). Similarly, archaeal RIO kinases (*rio1* and *rio2*) are involved in the maturation of the small ribosomal subunit that arises from the intricate assembly of several ribosomal RNAs and ribosomal proteins (25), and *rio1* is required for the motility of *S. acidocaldarius* (22). The present results suggest an additional role for the archaeal RIO kinase *rio1* in the response of *S. acidocaldarius* to UV irradiation (22, 25) (Fig. 2 and 3). The eukaryotic-type response to DNA damage in archaea involving the use of protein phosphorylation as a signal has not yet been examined (56). Therefore, the present study is the first to demonstrate the involvement of protein kinase in the response of archaea to UV irradiation.

Saci_0951 and Saci_1302 were both strongly induced (11- and 7-fold, respectively) following UV irradiation (14). However, these genes encode hypothetical proteins. In the present study, the Saci_0951 and Saci_1302 knockout strains became sensitive to UV irradiation (Fig. 2 and 3). Since the sensitivity patterns of ΔSaci_0951 and ΔSaci_1302 to DNA damage are distinct (Table 1), we speculate that Saci_0951 and Saci_1302 participate in different cellular processes. However, we currently cannot clarify the reasons for the sensitivity of ΔSaci_0951 and ΔSaci_1302 to UV irradiation because a sequence motif (putative conserved domain) of these proteins was not identified. Unexpectedly, the hypothetical protein Saci_0951 knockout strain exhibited the strongest sensitivity to UV irradiation in the present study (Fig. 2).

The sensitivities of the knockout strains to DNA damage are summarized in Table 1. The knockout strains were not sensitive replication inhibitors (Table 1). However, Δ*cdc6-2*, Δ*rio1*, and ΔSaci_0951 exhibited sensitivities to a wide variety of helix-distorting DNA lesions, including UV-induced DNA damage, the intra-strand crosslink, and bulky adducts (Table 1). We concluded that Cdc6-2, RIO1, and Saci_0951 are important in the response or DNA repair of UV-induced DNA damage and helix-distorting DNA lesions. Similarly, the general activator TFB3 is required in the response to helix-distorting DNA lesions (Table 1). The results obtained revealed that UV-regulated genes are not specifically involved in the response to UV irradiation. In contrast, Saci_1302 may be specifically involved in this response (Table 1). We speculate that the repression of DNA replication by Cdc6-2 is important in the response to general DNA damage with potential for DSB rather than in the specific response to helix-distorting DNA lesions. UV-induced damage, cisplatin, and 4-NQO induce DNA-less cell formation in *S. islandicus* (20). Sun *et al.* recently demonstrated that Orc1-2/Cdc6-2 of *S. islandicus* interacts with a conserved hexanucleotide motif present in UV-regulated gene promoters (28) and regulates their expression (48). *S. islandicus* Δ*cdc6-2* also showed hypersensitivity to 4-NQO treatment, indicating that it acts as key regulators in the DNA damage (4-NQO) response in *S. islandicus* (48). The results of our experiments and those of Sun et al. (48) suggest that Orc1-2/Cdc6-2 acts as a key regulator in helix-distorting DNA damage.

Helix-distorting DNA lesions are generally removed via nucleotide excision repair (NER). However, recent genetic studies did not identify a canonical NER pathway in hyperthermophilic archaea (13, 18, 57, 62). Furthermore, excision activity through NER using a crude extract in hyperthermophilic archaea has not yet been demonstrated (56). Therefore, the NER pathway in hyperthermophilic archaea remains unclear. Homologous recombination-mediated stalled fork DNA repair has been proposed as a possible repair pathway for helix-distorting DNA damage (13, 18). The present study identified the genes involved in the responses to helix-distorting DNA damage of *S. acidocaldarius*. However, further studies are required to elucidate the DNA repair mechanisms of helix-distorting DNA lesions. Moreover, future investigations on the RIO kinase and the hypothetical protein Saci_0951 may provide insights into the helix-distorting DNA repair pathway in this archaeon. The RIO kinase may potentially modulate the activity of DNA repair proteins, and Saci_0951 may directly or indirectly participate in helix-distorting DNA repair.

We noted that the replacement of *rio1* and Saci_0951 with a *pyrE-lacS* marker cassette may attenuate the genes encoded downstream of the marker cassette (Saci_0966 [RNA-processing protein] in Δ*rio1*, Saci_0949 [hypothetical protein], and Saci_0950 [GHMP kinase] in ΔSaci_0951) in the same putative operons (Fig. 1B). We performed knockout experiments on Saci_0966, Saci_0949, and Saci_0949–0950 (triplicates), which did not reveal the growth of transformant colonies. We speculate that these genes are essential, which suggests that no attenuation of Saci_0966 in DP-8 (Δ*rio1*) or Saci_0949 and Saci_0950 in DP-9 (ΔSaci_0951) occurred.

The members of the order *Sulfolobales* inhabit an extreme habitat; however, the underlying molecular mechanisms to tolerate sunlight were not previously examined. The present study revealed that *S. acidocaldarius* utilizes common genes for its response to UV-induced damage and other types of helix-distorting DNA damage. The present results may assist us to envision and subsequently explain the mechanisms through which *Sulfolobales* respond to and overcome DNA damage.

## SUPPLEMENTARY MATERIAL



## Figures and Tables

**Table 1:** Sensitivities of knockout stains to DNA damage.

	Type of DNA damage	DP-1	Δ*cdc6-2*	Δ*tfb3*	Δ*rio1*	ΔSaci_0951	ΔSaci_1302
UV	CPD	−	++	+	+	++	+
Cisplatin	Intra-strand crosslink^a^	−	++	−	+	++	−
4-NQO	Bulky adduct^a^	−	++	+	+	+	−
Metronidazole	Bulky adduct^a^	−	++	+	++	++	+
Actinomycin D	Inhibition of the elongation stage of replication	−	−	−	−	−	−
Novobiocin	Reduction in the replication rate	−	−	−	−	−	−

The sensitivities of knockout strains to DNA damage are summarized. −, +, and ++ indicate no sensitivity, sensitivity, and strong sensitivity, respectively. No sensitivity means that the sensitivity of the knockout strain is the same as that of the parent strain.

a Sakofsky *et al.* (41).
