# Supplementary material for: Participation of UV-regulated Genes in the Response to Helix-distorting DNA Damage in the Thermoacidophilic Crenarchaeon Sulfolobus acidocaldarius
Source: Microbes Environ. 2019 Dec 27;34(4):363–73. doi: 10.1264/jsme2.ME19055 (PMC6934391; doi:10.1264/jsme2.ME19055)
Supplement: Supplementary file 1 [file 34_363_s1.pdf]

Participation of UV-Regulated Genes in the Response to Helix-Distorting DNA Damage in the Thermoacidophilic Crenarchaeon Sulfolobus acidocaldarius

Shoji Suzuki and Norio Kurosawa*

Supplemental table 1 Strains or DNA used in this study.

Strains or plasmids	Relevant characteristic(s)	Source or reference
Strains		
S. acidocaldarius		
SK-1	MR31 (18) with Δ*suaI* (Δ*pyrE* Δ*suaI*)	(49)
DP-1	SK-1 with Δ*phr* (Δ*pyrE* Δ*suaI* Δ*phr*)	(50)
DP-6	DP-1 with Δ*cdc6-2*::*pyrE-lacS* (Δ*suaI* Δ*phr* Δ*cdc6-2*::*pyrE-lacS*)	This study
DP-7	DP-1 with Δ*tfb3*::*pyrE-lacS* (Δ*suaI* Δ*phr* Δ*tfb3*::*pyrE-lacS*)	This study
DP-8	DP-1 with Δ*rio1*::*pyrE-lacS* (Δ*suaI* Δ*phr* Δ*rio1*::*pyrE-lacS*)	This study
DP-9	DP-1 with ΔSaci_0951::*pyrE-lacS* (Δ*suaI* Δ*phr* ΔSaci_0951::*pyrE-lacS*)	This study
DP-10	DP-1 with ΔSaci_1302::*pyrE-lacS* (Δ*suaI* Δ*phr* ΔSaci_1302::*pyrE-lacS*)	This study
Plasmids		
placSpyrE	Plasmid DNA carrying 800 bp of 5' and 3' homologous regions of suaI locus at both ends of *pyrE-lacS* dual marker	(50)
PCR product		
Cdc6-2-knock	Linear DNA carrying 48 bp of 5' and 3' homologous regions of cdc6-2 locus at both ends of *pyrE-lacS* dual marker	This study
TFB3-knock	Linear DNA carrying 48 bp of 5' and 3' homologous regions of tfb3 locus at both ends of *pyrE-lacS* dual marker	This study
RIO-knock	Linear DNA carrying 48 bp of 5' and 3' homologous regions of rio1 locus at both ends of *pyrE-lacS* dual marker	This study
Saci_0966-knock	Linear DNA carrying 38 bp of 5' and 3' homologous regions of Saci_0966 locus at both ends of *pyrE-lacS* dual marker	This study
Saci_0951-knock	Linear DNA carrying 48 bp of 5' and 3' homologous regions of Saci_0951 locus at both ends of *pyrE-lacS* dual marker	This study
Saci_0949-knock	Linear DNA carrying 38 bp of 5' and 3' homologous regions of Saci_0949 locus at both ends of *pyrE-lacS* dual marker	This study
Saci_0949 –0950-knock	Linear DNA carrying 38 bp of 5' and 3' homologous regions of Saci_0950 and Saci_0949 loci at both ends of *pyrE-lacS* dual marker, respectively	This study
Saci_1302-knock	Linear DNA carrying 48 bp of 5' and 3' homologous regions of Saci_1302 locus at both ends of *pyrE-lacS* dual marker	This study

Supplemental Table 2 Primers used in this study.

Primers	Sequence[a] (5’-3’)
Cdc6-2-KO-F	AAGTCTCTGGGTCAAAATATATAGTATTACATCTAAAAATAGGATTAG**TGTTTTTCTCTATATCAATCTC**
Cdc6-2-KO-R	CTTTTAAAATAACGGAGTTTATGAGATCCTCGATTGTTCTAACCTCTT**ACTCCTAGATCTAAAACTAAAG**
Cdc6-2-out-F	GAGAAATTGAAATAGGCATAG
Cdc6-2-out-R	TCCTTATTAGTTTAGTCTTATC
TFB3-KO-F	ACATTTGTCAAACAACGTAAAAATATTTTTCAGGAGATTTTTTGTTCT**TGTTTTTCTCTATATCAATCTC**
TFB3-KO-R	TCATAAGAAGAGGTTATATAAAAATTTTTCCATCTATAAAAATCATTT**ACTCCTAGATCTAAAACTAAAG**
TFB3-out-F	GTTCTCGTTAAGAACCATTAC
TFB3-out-R	GTCCGTATGTTGTTTTTAGTG
RIO-KO-F	ACAAGGTCATCAGCCAGGATGTGATAGAACAGCTCAGAGGATAATTCT**TGTTTTTCTCTATATCAATCTC**
RIO-KO-R	ACATCATAGTTACTCATATTAAATTTTAATAAAACGAGTTAATATTTC**ACTCCTAGATCTAAAACTAAAG**
RIO-out-F	TACTGATGGAAAAGAGAGAC
RIO-out-R	TATTACCGATATGACTTTCATG
Saci_0966-KO-F	TTAACTCGTTTTATTAAAATTTAATATGAGTAACTATG**TGTTTTTCTCTATATCAATCTC**
Saci_0966-KO-R	TCCTTTAAATTTCTATTAACGTTAGATAACATAGCGTA**ACTCCTAGATCTAAAACTAAAG**
Saci_0951-KO-F	CATTTACCTCTTCCTCCGTTATAACTCAATAAGAGTAATATGGATGAT**TGTTTTTCTCTATATCAATCTC**
Saci_0951-KO-R	ATAAAAAGGATTGGAATATTATATTAATTTATACATCATGTCCTCGTG**ACTCCTAGATCTAAAACTAAAG**
Saci_0951-out-F	ACTAATATTACAATCCTAACTG
Saci_0951-out-R	CAGCTTTGTTTGATCTCTTTC
Saci_0949-KO-F	TTCAT**TGA**TACCAGAGAATCATCCCAGAAGAGAGTCAC**TGTTTTTCTCTATATCAATCTC**
Saci_0949 –0950-KO-F	TGTATAAATTAATATAATATTCCAATCCTTTTTATAGC**TGTTTTTCTCTATATCAATCTC**
Saci_0949 –0950-KO-R	CTTTCCGATATGAATCTTAGGGATTCTTTTAAAATTTC**ACTCCTAGATCTAAAACTAAAG**
Saci_1302-KO-F	CGTTACTTTCGCTCATCACCATCTGTTTATTAAAACAAGAATTCAAAC**TGTTTTTCTCTATATCAATCTC**
Saci_1302-KO-R	TTTGGATCCATAACGGGTTAAGTGAAAAATAAGAATAAAAATAATGTC**ACTCCTAGATCTAAAACTAAAG**
Saci_1302-out-F	GTAAAATCTACACTCACTTCAC
Saci_1302-out-R	AGTATCAATTGAACTAGCTATG

[a] 5’ and 3’ homologous regions of target gene are underlined, and sequences of KO primers that anneal with the *pyrE-lacS* marker genes are in **bold**.
